# Immunohistochemistry Helps to Distinguish Noninvasive Follicular Thyroid Neoplasm with Papillary-like Nuclear Features/Noninvasive Encapsulated Follicular Variant of Papillary Thyroid Carcinoma with Other Follicular Thyroid Lesions

**DOI:** 10.3390/medicina57111246

**Published:** 2021-11-14

**Authors:** Hao-Wen Chuang, Jyh-Seng Wang, Jen-Wei Tsai, Chao-Tien Hsu, Kai-Jen Lin

**Affiliations:** 1 Department of Pathology, E-Da Hospital, I-Shou University, Kaohsiung 82445, Taiwan; hwchuang1980@gmail.com (H.-W.C.); jwtsai0317@gmail.com (J.-W.T.); ed103797@edah.org.tw (C.-T.H.); 2 Department of Pathology and Laboratory Medicine, Kaohsiung Veterans General Hospital, Kaohsiung 81314, Taiwan; jswang@vghks.gov.tw; 3 Institute of Oral Biology, School of Dentistry, National Yang Ming Chiao Tung University, Taipei 11221, Taiwan

**Keywords:** immunohistochemistry, *BRAF* ^V600E^, NIFTP, encapsulated, follicular variant, papillary thyroid carcinoma, differential diagnosis

## Abstract

*Background and Objectives:* We aimed to assess the diagnostic value of various immunohistochemical (IHC) markers and panels for differentiation among benign follicular nodules (BFNs), noninvasive follicular thyroid neoplasms with papillary-like nuclear features (NIFTPs), noninvasive encapsulated follicular variants of papillary thyroid carcinoma (NEFVPTCs), and infiltrative FVPTC (IFVPTC). *Materials and Methods:* Sixty-three cases were classified as BFNs, NIFTPs, NEFVPTCs, or IFVPTCs and were evaluated using the following markers: CK19, CD56, galectin-3, CITED1, HBME-1, VE1, and TROP-2. *Results:* The IHC results for NIFTP and NEFVPTC exhibited no statistically significant differences. In differentiating IFVPTCs from BFNs and NIFTPs/NEFVPTCs, galectin-3 and TROP-2 were the markers with the highest sensitivity plus high specificity, respectively. In various combinations, panel co-expression of two markers, including galectin-3 and/or HBME-1 and/or TROP-2, and the combination of galectin-3 and TROP-2 co-expression could achieve 100% in all aspects. In terms of discrimination of BFNs from NIFTP/NEFVPTC, CK19 was the single most sensitive marker (81.3%), while CD56 was the most specific (100%). The panel consisting of CK19 and/or HBME-1 exhibited the greatest sensitivity (96.9%), but the panel with CD56 and/or HBME-1 exhibited the greatest specificity (90.5%). *Conclusions:* Our results broaden the use of IHC markers for differential diagnoses among the four groups of follicular-based lesions. In addition, the similar IHC profiles of NIFTP and NEFVPTC also suggest the original criterion of <1% papillae within tumors, providing a reliable NIFTP diagnosis. Their close relationship may represent a spectrum of progressing neoplasia.

## 1. Introduction

Thyroid carcinomas are prime examples of intensified surveillance resulting in an increased incidence of early cancers exhibiting indolent behavior. This phenomenon is commonly described as cancer overdiagnosis, and is mainly attributable to the enhanced screening of papillary thyroid carcinoma (PTC), comprising ~80% of thyroid epithelial malignancies, particularly the follicular variant of PTC (FVPTC). Studies have demonstrated that FVPTC and its subtype, encapsulated FVPTC (EFVPTC), exhibit indolent behavior and are genetically distinct from infiltrative tumors. Owing to the recognition of overdiagnosis and overtreatment in indolent cancers of the thyroid, an international multidisciplinary study proposed new terminology—noninvasive follicular thyroid neoplasm with papillary-like nuclear features (NIFTP)—for noninvasive EFVPTC and established reproducible criteria [1].

The accurate differentiation of NIFTP and other follicular-based lesions, such as FVPTC and follicular adenoma (FA), benefits patients undergoing an operation, and the subsequent surveillance and research [2,3].

The criteria initially proposed for NIFTP were well defined, and the indolent behavior of NIFTP has been observed in some studies [4,5]; however, the criteria application was subjective [6] and lymph node or distant metastases were observed in some cases despite strict criteria being applied [7,8,9]. Therefore, refinement criteria were proposed but were generally not accepted as their use resulted in increased workload [10,11,12,13,14]. As follicular-patterned thyroid neoplasms, including NIFTP, often harbor RAS mutations or RAS-like mutations, and almost never BRAFV600E mutations, the presence of which can exclude NIFTP, molecular testing may be time-consuming and expensive [15,16]. Moreover, the diagnosis of PTC via histologic evaluation has proved challenging, particularly in differentiating between FVPTC and other follicular-patterned thyroid neoplasms. Several studies have been conducted to resolve problems regarding the application of immunohistochemical (IHC) markers, such as cytokeratin 19 (CK19), CD56, galectin-3, CBP/p300-interacting transactivator 1 (CITED1), Hector Battifora mesothelial cell 1 (HBME-1), anti-BRAFV600E (VE1) antibody, and trophoblast cell-surface antigen-2 (TROP-2) [14,16,17,18,19,20,21,22]. Conversely, IHC studies of NIFTP were still limited for several markers and there was no comparison of the panel in the application of differential diagnosis [13,14,17,22,23,24,25].

The purpose of this study was to evaluate the diagnostic value of a panel, including CK19, CD56, galectin-3, CITED1, HBME-1, VE1, and TROP-2, for differentiation among benign follicular nodules (BFNs), NIFTPs, and FVPTCs.

## 2. Materials and Methods

### 2.1. Case Selection

During 2009–2017, surgically resected thyroid lesions were retrospectively reviewed from the archive of the pathology department at E-Da Hospital. The E-Da Hospital ethical committee approved this study (approval code: EMRP-105-078). Histopathological reports were reviewed. Study groups were created as follows: BFN, NIFTP, and FVPTCs, including noninvasive EFVPTCs (NEFVPTCs) and infiltrative FVPTCs (IFVPTCs).

BFNs included FAs, Hürthle cell adenomas (HAs), and hyperplastic nodules (HDs). The diagnosis of FAs and HAs was based on the diagnostic criteria of the 4th World Health Organization (WHO) classification system [26,27]. HD diagnosis was based on the presence of variable-sized follicles containing colloid and lined by bland follicular cells without crowding, overlapping nuclei, or other PTC-type nuclei. NIFTP was diagnosed according to the reported criteria defined by Nikiforov et al. [1,28] and other studies [10,11,12,29]. PTC diagnosis was also based on the criteria of the 4th WHO classification system, including nuclear irregularity (grooves, indentations, clearing, and increased size) and pseudoinclusions, and tumors composed completely or almost entirely (99% of the tumor) of follicles lined by cells with adequate nuclear features were confirmed as FVPTC [30,31]. FVPTCs were subdivided into EFVPTCs and IFVPTCs. FVPTCs with complete encapsulation and presence of <1% papillary formations were subtyped as EFVPTC in our study. We only collected cases without true capsular or vascular invasion, or NEFVPTCs, for comparison [30]. FVPTCs without a complete tumor capsule and with tumor tongues infiltrating the thyroid parenchyma or diffuse growth patterns were subtyped as IFVPTCs [22,31,32,33].

The histological diagnosis for each case was reviewed, and cases for which a consensus diagnosis was achieved by 2 pathologists (H.-W.C. and K.-J.L.) were used. In total, 63 cases were selected for the study, comprising 21 BFNs (8 FAs, 5 HAs, and 8 HDs), 16 NIFTPs, 16 NEFVPTCs, and 10 IFVPTCs.

### 2.2. Immunohistochemical Staining

The IHC procedures, conducted using a BOND-MAX Autostainer, were based on the manufacturers’ recommendations (Leica Microsystems, Bannockburn, IL, USA). IHC staining was performed on whole representative sections (3 µm thick) from each archival tissue block after antigen retrieval in ethylenediaminetetraacetic acid buffer at pH 9.0 for 20 min. The sections were incubated with primary antibodies against CK19, CD56, galectin-3, CITED1, HBME-1, VE1, or TROP-2 at room temperature for 30 min; with a bond polymer refine detection kit using postprimary and polymer reagent for 8 min; with 3,3-diaminobenzidine for 10 min; and with hematoxylin as the counterstain for 5 min using CD56 (1:500), CK19 (1:400), and CITED1, HBME-1 and galectin-3 (1:200), TROP-2 (1:50), and VE1 (1:100). The following primary monoclonal antibodies were used: CD56 antibody (clone RCD56; Zytomed, Berlin, Germany), CK19 antibody (clone B170; Leica, Newcastle, UK), HBME-1 antibody (clone HBME-1; Genemed, South San Francisco, CA, USA), galectin-3 antibody (clone 9C4; Leica, Bannockburn, IL, USA), CITED1 (clone CITED-1; Abcam, Cambridge, UK), TROP-2 antibody (clone F-5; Santa Cruz Biotechnology, Dallas, TX, USA), and anti-*BRAF*^V600E^ (VE1) antibody (clone VE1; Spring Bioscience, Pleasanton, CA, USA). Slides were examined using an Eclipse 80i optical microscope (Nikon, Tokyo, Japan) with NIS-Elements D digital imaging software (Nikon Instruments Inc., Melville, NY, USA). Intraductal carcinoma of the breast for CK19; pancreas for CD56; normal breast tissue for CITED1; a classic PTC known to react with galectin-3, HBME-1, and VE1; and placental tissue for TROP-2 were used as the positive controls. PBS was used as the negative control instead of the primary antibody.

### 2.3. Immunohistochemical Evaluation

CK19, CD56, HBME-1, galectin-3, CITED1, VE1, and TROP-2 staining exhibited cytoplasmic or membranous expression, membrane staining, membrane staining along the lateral and abluminal surfaces ± cytoplasmic, cytoplasmic and nuclear staining, nuclear and cytoplasmic expression, cytoplasmic expression, and membranous expression, respectively. A cell-staining ≥50% was considered diffusely positive; staining of <50% was considered focally positive. For the aforementioned markers, except for TROP-2, a lesion was considered positive when >10% of the cells exhibited specific antibody reactivity. For TROP-2, membranous staining of >5% of the cells were considered positive [15,16,17,18,19]. The results of IHC were assessed by 2 pathologists (H.-W.C. and K.-J.L.) and a consensus regarding controversial cases was achieved using a multiheaded microscope.

### 2.4. Statistical Analysis

Statistical analyses were performed using MedCalc for Windows, version 19.1.6 (MedCalc Software, Ostend, Belgium). Associations between categorical variables were evaluated using Fisher’s exact test or the chi-square test as appropriate, and a 2-tailed *p*-value < 0.05 was considered statistically significant. In addition, sensitivity, specificity, positive predictive value (PPV), negative predictive value (NPV), and diagnostic accuracy were calculated using traditional formulae for diagnostic tests.

## 3. Results

Table 1 shows a summary of the clinical, pathological, and immunohistochemical features of each marker in each group of thyroid lesions.

### 3.1. Clinicopathological Features

This cohort featured 21 BFNs (8 FAs, 5 HAs, and 8 HDs), 16 NIFTP, 16 NEFVPTC, and 10 IFVPTC cases. A significant female predominance was observed for all groups. The female:male ratios were 16:5, 12:4, 15:1, and 8:2 in BFN, NIFTP, NEFVPTC, and IFVPTC groups, respectively. The mean ages at the time of presentation within BFN, NIFTP, NEFVPTC, and IFVPTC groups were 44.0, 50.9, 37.1, and 43.6 years, respectively. The mean lesion sizes were 2.42 (range 0.7–4.8), 2.40 (range 0.2–6), 2.26 (range 0.5–3.2), and 1.68 (range 1.1–2.6) cm in BFN, NIFTP, NEFVPTC, and IFVPTC groups, respectively. Multifocality was noted in BFN, NEFVPTC, and IFVPTC groups, and the ratios were 8:21 (all HDs), 2:16, and 4:10, respectively. Four of the IFVPTC cases were associated with extrathyroidal extension and four had lymph node metastases. No distant metastasis was observed. The median follow-ups for BFN, NIFTP, NEFVPTC, and IFVPTC groups were 41.5 (range 1.0–102), 45.0 (range 1.0–91.0), 47.5 (range 1.0–98.0), and 70.0 (range 1.0–87.0) months, respectively (Table 1).

### 3.2. Immunohistochemical Features

#### 3.2.1. BFNs

**Figure 1:**
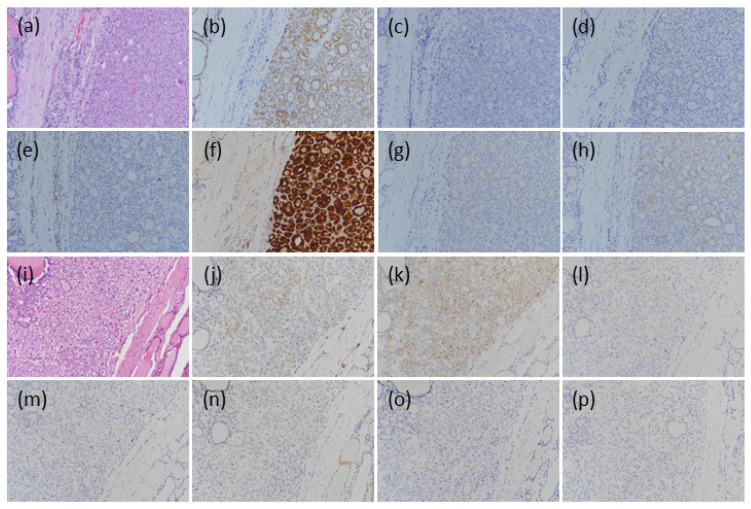
Representative microscopic findings for expression of the seven markers in benign follicular nodules and noninvasive follicular thyroid neoplasm with papillary-like nuclear features (NIFTP). (**a**) Hematoxylin–eosin (HE) stain for a Hürthle cell adenoma; (**b**) CD56 showed diffuse and complete membrane staining; (**c**) CK (cytokeratin) 19 and (**d**) HBME-1 showed no expression; (**e**) galectin-3 showed focal cytoplasmic staining; (**f**) CITED1 showed diffuse cytoplasmic and nuclear staining; (**g**) VE1 showed negative staining; (**h**) TROP-2 showed focal staining, but the majority of follicular adenomas showed negative staining; (**i**) HE stain for a NIFTP; (**j**) CD56 showed diffuse staining; (**k**) CK19 showed diffuse staining; (**l**) HBME-1 showed negative staining in this case but more than half of the NIFTPs showed positive results; (**m**) galectin-3 showed negative staining; (**n**) CITED1 showed focal cytoplasmic and nuclear staining in this case, consistent with a negative result. Most NIFTP cases, however, showed positive results; (**o**) VE1 and (**p**) TROP-2 showed no expression (original magnification, 200×).

All BFNs were positive for CD56, with diffuse expression in 19 of 21 cases, and focal expression in the other two, one of which was an FA, and the other an HD. For CK19, four of 21 (19.0%) cases were positive: three exhibited focal staining, two were FAs, and one was an HA, with the FA showing diffuse staining. HBME-1 was positive in two cases (9.5%), and both were FAs. One case exhibited focal staining and the other diffuse staining. Galectin-3 was positive in only one case (4.8%), an FA, which showed diffuse staining. CITED1 was positive in 12 of 21 (57.1%) cases, among which nine exhibited diffuse staining. For VE1, expression was absent in all the cases. TROP-2 was negative in the majority of the cases, but four cases—one FA and three HAs—resulted in a focal staining pattern (Table 1; Figure 1a–h).

#### 3.2.2. NIFTP

For CD56, 13 out of 16 (82.4%) cases were positive, and 11 showed diffuse staining. For CK19, 12 of 16 (75.0%) cases were positive, and 11 showed focal staining and one showed diffuse staining. HBME-1 was positive in 9 of 16 cases (56.2%) with focal expression. Galectin-3 was positive in only one case (6.3%) with focal expression. CITED1 was positive in 11 out of 16 (68.8%) cases, and eight showed diffuse staining and three cases showed focal staining. VE1 expression was absent in all the cases. TROP-2 was positive in only one case (6.3%), and showed diffuse staining (Table 1; Figure 1i–p).

#### 3.2.3. NEFVPTC

**Figure 2:**
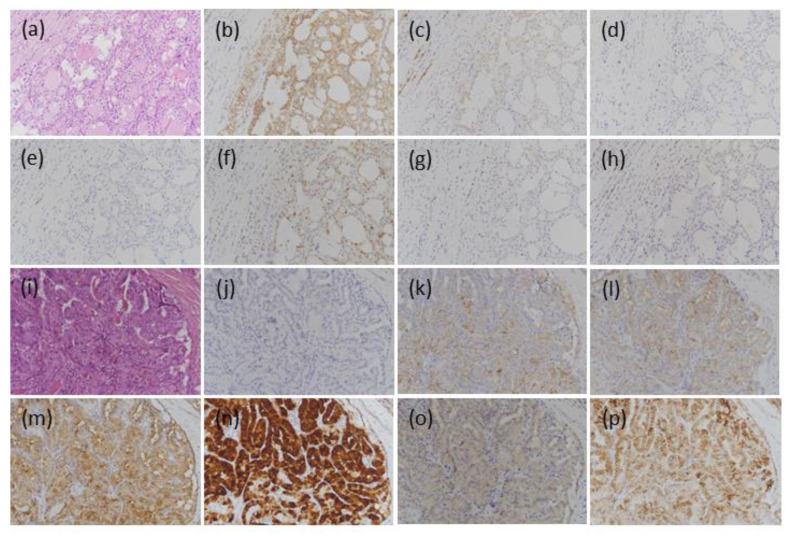
Representative microscopic findings for expression of the seven markers in noninvasive encapsulated follicular variants of papillary thyroid carcinomas (NEFVPTCs) and infiltrative follicular variants of papillary thyroid carcinomas (IFVPTCs). (**a**) HE stain for a NEFVPTC; (**b**) CD56 showed diffuse membrane staining; (**c**) CK19 showed focal cytoplasmic staining; (**d**) HBME-1 showed negative staining in this case, but more than half of the NEFVPTCs showed positive results; (**e**) galectin-3 showed negative staining; (**f**) CITED1 showed diffuse cytoplasmic and nuclear staining; (**g**) VE1 and (**h**) TROP-2 showed results of negative staining; (**i**) HE stain for an IFVPTC; (**j**) CD56 showed loss of expression; (**k**) CK19 showed diffuse cytoplasmic and membrane staining; (**l**) HBME-1 showed diffuse membrane staining; (**m**) galectin-3 showed diffuse cytoplasmic staining; (**n**) CITED1 showed diffuse cytoplasmic and nuclear staining; (**o**) VE1 showed homogenous cytoplasmic staining; (**p**) TROP-2 showed diffuse membrane staining (original magnification, 200×).

For CD56, 12 out of 16 (75.0%) cases were positive, seven showed focal expression, and the other five showed diffuse staining. For CK19, 14 out of 16 (87.5%) cases were positive, half of which showed focal staining and the others showed diffuse staining. HBME-1 was positive in 9 out of 16 cases (56.2%), and six showed diffuse staining and the other three showed focal expression. Galectin-3 was positive in only two cases (12.5%). One showed diffuse staining and the other focal staining. CITED1 was positive in 11 out of 16 (68.8%) cases, and ten showed diffuse staining and the rest focal expression. VE1 was positive in only one case (6.3%) with diffuse expression. TROP-2 was also positive in only one case (6.3%) with focal expression (Table 1; Figure 2a–h).

#### 3.2.4. IFVPTC

CD56 was positive in 1 of 10 cases (10.0%), and one showed diffuse expression. All the cases were positive for CK19, HBME-1, galectin-3, CITED1, and TROP-2. Of the CK 19-positive cases, six showed diffuse staining and four showed focal staining. For HBME-1-positive cases, six showed diffuse staining and four showed focal staining. Eight galectin-3-positive cases showed diffuse staining and two showed focal staining. All CITED1-positive cases showed diffuse expression. TROP2-positive cases showed diffuse staining in six cases and focal staining in four cases. VE1 was positive in four cases (40.0%) with diffuse expression (Table 1; Figure 2i–p).

Table 2, Table 3, Table 4 and Table 5 show a comparison of the seven immunohistochemical markers, alone or in combination with each group of thyroid lesions.

### 3.3. Comparisons between Various Lesions Using Single Markers

As shown in Table 2, the expression of the markers between BFNs and IFVPTC were all statistically significant (*p* < 0.05). Although all the CK19-positive cases were FAs (four of 13, 30.8%), the difference in expression for NIFTP and NEFVPTC was statistically significant (*p* = 0.0193). NIFTP and NEFVPTC both showed similar expression for all markers and the same result in a comparison with BFNs and IFVPTC. Statistical significance (*p* < 0.05) was achieved for all markers except for CK19 and CITED1 in the comparison of expression between the two lesions and IFVPTC. Only for CD56, CK19, and HBME-1 were the results of the expression comparison between the two lesions and BFNs statistically significant (*p* < 0.05). Owing to the similar immunophenotypes of NIFTP and NEFVPTC, we grouped them for the subsequent comparison and chose the markers with statistically significant expression in the comparison of lesions to evaluate the application of these markers used alone or in various panels for the differential diagnosis. Although CITED1 showed no statistical significance when NIFTP and NEFVPTC were compared with IFVPTC, separately, it showed a trend (*p* = 0.0538). Hence, CITED1 was also chosen to compare NIFTP/NEFVPTC with IFVPTC (*p* = 0.0330).

### 3.4. Diagnostic Application between BFNs and IFVPTC

As single antibodies, all markers except CD56 and VE1 achieved 100% sensitivity and NPV in our research; however, both CD56 and VE1 were the most specific markers and achieved 100% specificity and PPV. Although CITED1 achieved 100% sensitivity, it was the least specific marker, with only 42.9% specificity. Galectin-3 had the highest diagnostic accuracy and specificity (96.8% and 95.2%, respectively). CK19, HBME-1, and TROP-2 also achieved 100% sensitivity, as well as high specificity and diagnostic accuracy. In various combinations, the most notable results were the panels’ co-expression of two markers, including galectin-3, HBME-1, and TROP-2, for which 100% sensitivity, specificity, PPV, NPV, and diagnostic accuracy could be achieved (Table 3).

### 3.5. Diagnostic Application between NIFTP/NEFVPTC and IFVPTC

For a single marker, all markers except CD56 and VE1 exhibited 100% sensitivity and NPV. VE1 was the most specific marker, with 96.9% specificity and 80.0% PPV. TROP-2 had the next best specificity (93.8%) and was the marker with the greatest diagnostic accuracy (95.2) and PPV (83.3%). This was followed by galectin-3, with 92.9% diagnostic accuracy, 90.6% specificity, and 76.9% PPV. Although HBME-1 and CITED1 achieved 100% sensitivity, their specificities (43.8% and 31.3%, respectively) and PPVs (35.7% and 31.3%, respectively) were low. In the aforementioned markers, the combination of galectin-3 and TROP-2 co-expression could achieve 100% sensitivity, specificity, PPV, NPV, and diagnostic accuracy (Table 4).

### 3.6. Diagnostic Application between BFNs and NIFTP/NEFVPTC

As shown in Table 5, for a single marker, only CD56, CK19, and HBME-1 exhibited statistically significant differences in expression. CK19 was also the most sensitive of the markers, with 81.3% sensitivity, 81.0% specificity, 86.7% PPV, 73.9% NPV, and the greatest diagnostic accuracy of up to 81.1%. HBME-1 was the next most sensitive, with 56.3% sensitivity, 90.5% specificity, 90.0% PPV, 57.6% NPV, and 69.8% diagnostic accuracy. CD56 was the least sensitive marker, with only 21.9% sensitivity, 45.7% NPV, and 52.8% diagnostic accuracy, but it exhibited the greatest specificity and PPV of up to 100%. In various combinations, the panel comprising CK19 and/or HBME-1 exhibited the greatest sensitivity, NPV, and diagnostic accuracy (96.9%, 94.4%, and 88.9%, respectively), but the panel comprising CD56 and/or HBME-1 exhibited the greatest specificity and PPV (90.5% and 92.0%, respectively). The addition of a third marker to the immuno-panel (the association of CD56, CK19, and HBME-1) not only failed to improve the sensitivity but also lowered the specificity, NPV, and diagnostic accuracy (76.2%, 94.1%, and 88.7%, respectively).

## 4. Discussion

In this study, multiple markers (CD56, CK19, galectin-3, CITED1, HBME-1, VE1, and TROP-2) were used to assess the expression and possible diagnostic role in BFN, NIFTP, NEFVPTC, and IFVPTC cases. Recently, Xu et al. [13] found that for patients with encapsulated or well-circumscribed tumors with a papillae proportion of <1%, no lymph node metastases developed, irrespective of their invasive status. The authors claimed that the original criterion of <1% papillae within the tumor remained sound for a diagnosis of NIFTP. The IHC results for NIFTP and NEFVPTC in our study were similar and exhibited no statistically significant differences. It remains consistent with the findings of the study by Xu et al. and suggests similar biological behaviors between NIFTP and NEFVPTC (previous NIFTP with <1% papillae). Although the only case of NEFVPTC in our study with a positive result for VE1 may require deeper sections in paraffin blocks to identify more papillae owing to the similar IHC findings obtained, both NIFTP and NEFVPTC were grouped for comparison with BFNs and IFVPTC using the aforementioned markers, both alone and in various combinations to determine their diagnostic value.

CD56 is a membrane glycoprotein with prominent roles in cell–cell adhesion [34] and the loss of CD56 expression has been correlated with tumor progression and poor prognosis in patients with some malignant tumors, including PTC [35]. Our study showed that CD56 was positively stained in all BFNs and lacked expression in 90.0% of IFVPTC cases. For NIFTP, CD56 staining was absent in 85% of cases from Tastekin et al. [23] and in 60% of cases from Cho et al. [22]. The positivity rate was higher in our study. CD56 was positively stained in 82.4% and 75.0% of cases with NIFTP and NEFVPTC, respectively. CD56 alone can distinguish PTC from not only BFNs but also NIFTP/NEFVPTC. Although it was observed to be the least sensitive of the markers (21.9%) in distinguishing BFNs from NIFTP/NEFVPTC, the PPV was 100%.

CK19, a low-molecular-weight cytokeratin, has been demonstrated to exhibit consistent overexpression in PTC; however, it is also known to provide positive staining results in benign nodules and FA [36]. In our study, CK19 was positively stained in 19.0%, 75.0%, 87.5%, and 100% of cases of BFN, NIFTP, NEFVPTC, and IFVPTC, respectively. The expression of CK19 in BFNs was significantly statistically different from that in NIFTP, NEFVPTC, and IFVPTC. For distinguishing malignant and benign lesions, in the study of Ma et al. [19], the sensitivity and specificity of CK19 were 100% and 56.25%, respectively. Following previous studies, the sensitivity and specificity of CK19 was 100% and 81.0% between BFNs and IFVPTC, and 81.3% and 81.0%, between BFNs and NIFTP/NEFVPTC, respectively. Consistent with Sadiq et al. [24], CK19 cannot differentiate IFVPTC from NIFTP/NEFVPTC, but it can be used to distinguish BFNs from IFVPTC, NIFTP, and NEFVPTC.

HBME-1, a mesothelioma marker, is applied in the diagnosis of malignant thyroid tumors [36]. We observed that HBME-1 individually had favorable sensitivity (100%) and specificity (90.5%) in distinguishing IFVPTC from BFNs but had relatively low specificity (100% sensitivity and 43.8% specificity) in distinguishing IFVPTC from NIFTP/NEFVPTC. Tastekin et al. [23] found no differences of expression in HBME-1 between NIFTP and benign groups. Nevertheless, Zargari et al. [17] showed two NIFTP cases and both expressed HBME-1, whereas only one case expressed HBME-1 in a total of 46 cases of the benign group. Cho et al. [22] also found statistically significant differences in the expression of HBME-1 between EFVPTC and follicular neoplasm. In our study, HBME-1 could distinguish BFNs from NIFTP/NEFVPTC with relatively low sensitivity (56.3%).

The other four markers, galectin-3, CITED1, VE1, and TROP-2, can be used to differentiate IFVPTC from BFNs and NIFTPs/NEFVPTCs. Galectin-3 is a beta-galactoside-binding animal lectin related to various mechanisms in tumor development and is believed to play a role in the malignant transformation of thyroid cells. Its expression has been noted in PTC, but some studies have also shown its expression in benign conditions and FAs [19]. CITED1 is the founding member of the CITED family of cofactors that are involved in regulating numerous CBP/p300-dependent transcriptional responses and plays a major role in the development and progression of PTC by promoting malignant cell proliferation through the activation of the Wnt/β-catenin signaling pathway [37]. *BRAF*^V600E^ mutations are frequently detected in classical PTCs, and a reliable and clinically validated antibody, VE1, has been developed to detect this mutation [14]. TROP-2, also known as tumor-associated calcium signal transducer 2, is a 35 kDa, 323-amino-acid, type-1 transmembranous glycoprotein, and its overexpression is present in the majority of human carcinomas, including thyroid carcinoma, and can serve as a potential diagnostic marker for PTC [17,38]. In distinguishing IFVPTC from BFNs and NIFTP/NEFVPTC, all of the markers except for VE1 achieved 100% sensitivity; however, VE1 exhibited the highest specificity and PPV for the differentiation of IFVPTC from BFNs (100%) and NIFTP/NEFVPTC (96.9%), respectively. Other studies revealed *BRAF*^V600E^ mutations in 20–80% of sporadic PTCs with a higher prevalence in conventional PTC than in follicular variants [15,16]. In the study of Johnson et al. [14], the expression of VE1 in NIFTP, invasive EFVPTC, and classical PTC with a predominantly follicular architecture was 0%, 14.6%, and 54.5%, respectively. Corresponding to the aforementioned studies, the results in our study suggest that VE1 was highly specific but insufficiently sensitive to distinguish IFVPTC from NIFTP/NEFVPTC. Among the other three markers with the highest sensitivity, galectin-3 and TROP-2 were the most specific markers in our study for differentiating IFVPTC from BFNs and NIFTP/NEFVPTC, respectively. Although Tastekin et al. [23] found statistically significant differences in the expression of galectin-3 between NIFTP and benign groups, the positive rate was low (15%) in NIFTP. Our study showed a low positive rate for both NIFTP/NEFVPTC (9.4%) and BFNs (4.8%) and no statistically significant differences, corresponding to the conclusion of Fu et al. [25], which also showed no significant difference between NIFTPs and benign lesions. Moreover, Zargari et al. [17] reported that TROP-2 could exhibit positive results in HAs. Excluding HAs, our study achieved a specificity of up to 93.8% in differentiating IFVPTC from BFNs.

Combined marker use was also tested to increase diagnostic value. For distinguishing IFVPTC from BFNs, the best panel was that of the co-expression of galectin-3 and/or HBME-1 and/or TROP-2; this aligned with the results of the previous studies [17,19,36,38]. Although Murtezaoglu et al. [39] showed different results, mainly in TROP-2, the discrepancy may have come from the difference in the study design, where Murtezaoglu et al. used tissue microarray for immunostaining, as well as the definition of FVPTC. For the differentiation of IFVPTC from NIFTP/NEFVPTC, the best panel would be that of the co-expression of galectin-3 and TROP-2, which can achieve 100% in all aspects of diagnostic value. Although Zargari et al. [17] examined two NIFTP cases, both positive for TROP-2, our study included 16 NIFTP cases, and the majority exhibited HBME-1 positivity, which was comparable with the results of Zargari et al. [17] and Johnson et al. [14]. To distinguish between NIFTP/NEFVPTC and BFNs, the panels comprising CK19 and/or HBME-1 exhibited the highest sensitivity (96.9%), and the panels comprising CD56 and/or HBME-1 exhibited the highest specificity (90.5%), accompanied by the lowest sensitivity (71.9%), among the three panels when two markers were used. The immunopanels with three markers not only failed to improve sensitivity but also lowered specificity. However, regardless of how many markers are used, immunopanels are better than single markers in differentiating NIFTP/NEFVPTC from BFNs when a patient exhibits questionable features fitting NIFTP criteria.

## 5. Conclusions

Our study is limited by the relatively small sample number and the absence of a correlation for molecular testing in this cohort; however, it possesses novelty and strength, and it broadens the use of IHC markers to differentiate IFVPTCs from BFNs, alone or in combination. Moreover, for borderline cases, highly sensitive and specific immunopanels, such as galectin-3 and/or HBME-1 and/or TROP-2 for IFVPTC and NIFTP/NEFVPTC and CK19 and/or HBME-1 for NIFTP/NEFVPTC and BFNs, can be useful. Using immunopanels, only borderline cases that are confirmed require additional sampling, and the diagnosis of tumors can be more accurately categorized for follow-up, treatment, and subsequent study. Finally, the similarity in immunohistochemical profiling between NIFTP and NEFVPTC in our study also indicates that the original criterion of <1% papillae within a tumor for a diagnosis of NIFTP remains sound. In addition, NIFTP is suggested to be an anticipated precursor of invasive EFVPTC [1,40]. The close relationship between NIFTP and NEFVPTC may represent a spectrum of progressing neoplasia. However, this is only a hypothesis and further study is required.

## Figures and Tables

**Table 1:** Summary of clinicopathological and immunohistochemical features in case groups.

	BFN	NIFTP	NEFVPTC	IFVPTC
Female/male	16/5	12/4	15/1	8/2
Mean age, years	44.0	50.9	37.1	43.6
Mean size, cm (range)	2.42 (0.7–4.8)	2.40 (0.2–6)	2.26(0.5–3.2)	1.68 (1.1–2.6)
Multifocality	8/21	0	2/16	4/10
Extrathyroidal extension	0	0	0	4/10
Lymph node metastases	0	0	0	4/10
Distant metastasis	0	0	0	0
FOP, months, median, (range)	41.5 (1.0–102)	45.0 (1.0–91.0)	47.5(1.0–98.0)	70.0 (1.0–87.0)
CD56 *	21/21 (100%)	13/16 (82.4%)	12/16 (75.0%)	1/10 (10.0%)
CK19	4/21 (19.0%)	12/16 (75%)	14/16 (87.5%)	10/10 (100%)
HBME-1	2/21 (9.5%)	9/16 (56.2%)	9/16 (56.2%)	10/10 (100%)
Gal-3	1/21 (4.8%)	1/16 (6.3%)	2/16 (12.5%)	10/10 (100%)
CITED1	12/21 (57.1%)	11/16 (68.8%)	11/16 (68.8%)	10/10 (100%)
VE1	0/21 (0%)	0/16 (0%)	1/16 (6.3%)	4/10 (40.0%)
TROP-2	4/21 (19.0%)	1/16 (6.3%)	1/16 (6.3%)	10/10 (100%)

BFN—benign follicular nodule; NIFTP—noninvasive follicular thyroid neoplasm with papillary-like nuclear features; NEFVPTC—noninvasive encapsulated follicular variant of papillary thyroid carcinoma; IFVPTC—infiltrative follicular variant of papillary thyroid carcinoma; FOP—follow up period; CK19—cytokeratin 19; Gal-3—galectin-3. * Positive staining for normal expression.

**Table 2:** The comparison for expression of seven immunohistochemical markers among different thyroid lesions.

IHC Markers	BFN vs. NIFTP	BFN vs. NEFVPTC	BFN vs. IFVPTC	NIFTP vs. NEFVPTC	NIFTP vs. IFVPTC	NEFVPTC vs. IFVPTC
CD56	*p* = 0.0412 *	*p* = 0.0167 *	*p* < 0.0001 *	*p* = 0.6738	*p* = 0.0005 *	*p* = 0.0016 *
CK19	*p* = 0.0008 * ^†^	*p* < 0.0001 * ^‡^	*p* < 0.0001 *	*p* = 0.3726	*p* = 0.0919	*p* = 0.2538
HBME-1	*p* = 0.0024 *	*p* = 0.0024 *	*p* < 0.0001 *	*p* = 1.0000	*p* = 0.0164 *	*p* = 0.0164 *
Gal-3	*p* = 0.8449	*p* = 0.3994	*p* < 0.0001 *	*p* = 0.5506	*p* < 0.0001 *	*p* < 0.0001 *
CITED1	*p* = 0.4768	*p* = 0.4768	*p* = 0.0156 *	*p* = 0.7141	*p* = 0.0538 ^#^	*p* = 0.0538 ^#^
VE1	*p* = 0.4111	*p* = 0.2519	*p* = 0.0023 *	*p* = 0.3173	*p* = 0.0070 *	*p* = 0.0372 *
TROP-2	*p* = 0.2658	*p* = 0.2658	*p* < 0.0001 *	*p* = 1.0000	*p* < 0.0001 *	*p* < 0.0001 *

BFN—benign follicular nodule; NIFTP—noninvasive follicular thyroid neoplasm with papillary-like nuclear features; NEFVPTC—noninvasive encapsulated follicular variant of papillary thyroid carcinoma; IFVPTC—infiltrative follicular variant of papillary thyroid carcinoma; CK19—cytokeratin 19; Gal-3—galectin-3. * Statistically significant (*p* < 0.05). ^†^ The comparison between follicular adenoma and NIFTP, *p* = 0.0193. ^‡^ The comparison between follicular adenoma and NEFVPTC, *p* = 0.0021. ^#^ The comparison between IFVPTC and NIFTP/NEFVPTC, *p* = 0.0330.

**Table 3:** The significance of the seven IHC markers, alone and in combinations, in BFN versus IFVPTC.

IHC Markers	Sensitivity (%)	Specificity (%)	PPV (%)	NPV (%)	Accuracy (%)
CD56	90.0	100	100	95.5	96.8
CK19	100	81.0	71.4	100	87.1
HBME-1	100	90.5	83.3	100	93.5
Gal-3	100	95.2	90.9	100	96.8
CITED1	100	42.9	45.5	100	61.3
VE1	40.0	100	100	77.8	80.6
TROP-2 *	100	81.0	71.4	100	87.1
Gal-3 and/or HBME-1 and/or TROP-2	100	100	100	100	100

BFN—benign follicular nodule; IFVPTC—infiltrative follicular variant of papillary thyroid carcinoma; CK19—cytokeratin-19; Gal-3—galectin-3; PPV—positive predictive value; NPV—negative predicting value. * For TROP-2, the sensitivity, specificity, PPV, NPV, and diagnostic accuracy in BFNs without Hürthle cell adenomas versus IFVPTCs was 100%, 93.8%, 90.9%, 100%, and 96.2%, respectively.

**Table 4:** The significance of the six markers, alone and in combinations, in NIFTP/NEFVPTC versus IFVPTC.

IHC Markers	Sensitivity (%)	Specificity (%)	PPV (%)	NPV (%)	Accuracy (%)
CD56	90.0	78.1	56.3	96.2	81.0
HBME-1	100	43.8	35.7	100	57.1
Gal-3	100	90.6	76.9	100	92.9
CITED1	100	31.3	31.3	100	47.6
VE1	40.0	96.9	80.0	83.8	83.3
TROP-2	100	93.8	83.3	100	95.2
Gal-3 and TROP-2	100	100	100	100	100

NIFTP—noninvasive follicular thyroid neoplasm with papillary-like nuclear features; NEFVPTC—noninvasive encapsulated follicular variant of papillary thyroid carcinoma; IFVPTC—infiltrative follicular variant of papillary thyroid carcinoma; CK19—cytokeratin-19; Gal-3—galectin-3; PPV—positive predictive value; NPV—negative predicting value.

**Table 5:** The significance of the three markers, alone and in combinations, in BFN versus NIFTP/NEFVPTC.

IHC Markers	Sensitivity (%)	Specificity (%)	PPV (%)	NPV (%)	Accuracy (%)
Single marker:					
CD56	21.9	100	100	45.7	52.8
CK19	81.3	81.0	86.7	73.9	81.1
HBME-1	56.3	90.5	90.0	57.6	69.8
Double markers:					
CK19 and/or HBME-1	96.9	77.3	86.1	94.4	88.9
CD56 and/or CK19	84.4	81.0	87.1	77.3	83.0
CD56 and/or HBME-1	71.9	90.5	92.0	67.9	79.2
Triple markers:					
CD56 and/or CK19 and/or HBME-1	96.9	76.2	86.1	94.1	88.7

BFN—benign follicular nodule; NIFTP—noninvasive follicular thyroid neoplasm with papillary-like nuclear features; NEFVPTC—noninvasive encapsulated follicular variant of papillary thyroid carcinoma; CK19—cytokeratin-19; PPV—positive predictive value; NPV—negative predictive value.

## Data Availability

The data used to support the findings of this study are available from the corresponding author upon request.
